# Pulmonary mucormycosis with bacterial coinfection in an adolescent with poorly controlled type 1 diabetes: a case report

**DOI:** 10.3389/fendo.2025.1724850

**Published:** 2026-01-08

**Authors:** Fu Rong, Zhang Mingying, Bai Ying, Huang Yiyun, Qian Ying, Xing Zheng, Chu Xiaolei

**Affiliations:** 1 Tianjin Children’s Hospital (Children’s Hospital, Tianjin University), Tianjin Key Laboratory of Birth Defects for Prevention and Treatment, Tianjin, China; 2 Tianjin Key Laboratory of Sports Physiology and Sports Medicine, College of Exercise & Health, Tianjin University of Sport, Tianjin, China; 3 Department of Rehabilitation, Tianjin University Tianjin Hospital, Tianjin, China

**Keywords:** adolescent patients, metagenomic sequencing, nebulized amphotericin B, pulmonary mucormycosis, type 1 diabetes

## Abstract

**Background:**

Pulmonary Mucormycosis (PM), a severe fungal infection affecting mainly immunocompromised individuals, is often caused by fungi like Rhizopus and Mucor. This report details a 12-year-old diabetic girl with pulmonary mucormycosis from an unusual Rhizopus species. Successful treatment involved stabilizing her blood glucose and managing multiple co-infections. This case provides important insights into diagnosing and treating rare fungal infections in diabetic children.

**Case summary:**

A 12-year-old girl with a two-year history of type 1 diabetes, inconsistently monitored, was hospitalized. She had a persistent cough for over ten days and a six-day high fever. Previous treatments with dexamethasone and antibiotics were ineffective. She showed symptoms of a productive cough, right-sided pleuritic chest pain, and a fever of 40°C. Examination revealed reduced breath sounds and moist rales in the right lung. Tests confirmed a severe infection, and imaging showed inflammatory consolidation, multiple cavitations, and pleural effusion in the right lung.

**Diagnosis:**

Metagenomic next-generation sequencing (mNGS) analyzes all nucleic acids from a patient’s bronchoalveolar lavage fluid to identify various pathogens without traditional cultures. The analysis identified Rhizopus species and Streptococcus pneumoniae, confirming pulmonary mucormycosis with a bacterial infection. Additionally, the glycated hemoglobin (HbA1c) level was 14.3%, indicating poorly controlled diabetes.

**Treatment:**

A comprehensive treatment regimen was employed. The bacterial co-infection was addressed with intravenous administration of meropenem and linezolid, while nebulized amphotericin B was utilized to treat the pulmonary mucormycosis. To mitigate the underlying risk factor, intensive glycemic control was achieved through the use of an insulin pump. Furthermore, bronchoscopy was conducted to clear respiratory secretions.

**Outcome:**

After 11 days in the hospital, the patient stabilized and was discharged. At a follow-up 1.5 months later, infection markers and blood glucose levels were normal.

**Conclusion:**

This case highlights the high risk of severe infections like pulmonary mucormycosis in adolescents with poorly managed type 1 diabetes. Metagenomic sequencing was crucial for quickly identifying co-infections. Successful treatment required a comprehensive approach, including targeted antimicrobial therapy, strict glycemic control, and bronchoscopic support, leading to a positive outcome.

## 1 Introduction

Pulmonary Mucormycosis (PM) is an invasive infection caused by fungi of the order Mucorales, with mortality rates as high as 50% to 80%. It is commonly seen in patients with diabetes, hematological malignancies, and those subjected to long-term immunosuppression (1). In recent years, the incidence of pulmonary mucormycosis in patients with diabetes has significantly increased, particularly among adolescents with poorly controlled blood glucose (HbA1c >8%). The underlying pathological mechanisms involve hyperglycemia-mediated immunosuppression, endothelial injury, and abnormal iron metabolism (2). However, such infections remain rare in the pediatric population, and early diagnosis is challenging, often being misdiagnosed as bacterial pneumonia or tuberculosis (3).

Traditional diagnostic methods rely on histopathology or cultures, but the fragile mycelium of Mucor species can easily be damaged during sample handling, leading to a positivity rate of less than 30% (4). The application of metagenomic next-generation sequencing (mNGS) technology has significantly improved the efficiency of pathogen detection, particularly in cases of mixed infections or low fungal load (5). In terms of treatment, liposomal amphotericin B remains the drug of choice; however, the optimization of dosing regimens (such as nebulization routes) and long-term management strategies in children require further evidence-based support (6).

This article reports on the diagnosis and treatment process of a 12-year-old patient with diabetes complicated by pulmonary mucormycosis, in which mNGS rapidly confirmed Rhizopus infection and nebulized amphotericin B combined with intensified insulin therapy achieved clinical stability. This case aims to emphasize: (1) the risk of invasive fungal infections in adolescent diabetes patients; (2) the value of mNGS in diagnosing rare pathogens; and (3) the necessity of multidisciplinary collaboration in managing complex infections.

## 2 Clinical data

### 2.1 General information

The patient is a 12-year-old female, admitted from January 7, 2025, to January 18, 2025, primarily due to a diagnosis of type 1 diabetes for over two years, a cough lasting more than ten days, and fever for six days eight days prior to admission. The patient received a diagnosis of diabetes at an external medical facility over two years prior. Approximately one year subsequent to this diagnosis, the patient was admitted to our hospital from September 26 to October 6, 2023, due to a diabetic ketoacidosis coma. To ascertain the specific type of diabetes, we performed islet autoantibody testing, which yielded negative results. An assessment of islet function revealed a significantly diminished intravenous C-peptide level of 0.073 nmol/L upon admission, indicating a severe impairment of pancreatic β-cell secretory function. Given the early age of onset, the presence of the characteristic “three polys and one less” symptoms at the onset, the sustained reliance on insulin therapy, and the objective laboratory evidence obtained during hospitalization indicating islet failure (as evidenced by extremely low C-peptide levels), the clinical presentation is consistent with type 1 diabetes. The patient has been receiving long-term treatment with aspart insulin (25 IU in the morning, 20 IU in the afternoon and evening) and detemir insulin (20 IU at bedtime), without regular blood glucose monitoring. Upon admission, the patient presented with a cough lasting for ten days (characterized by mucous and right-sided chest pain) and a fever lasting six days (with a peak temperature of 40°C). Prior outpatient treatment included dexamethasone, clindamycin, and ambroxol, which resulted in resolution of fever.

Immunization history includes BCG vaccine, poliovirus vaccine (for infantile paralysis), measles, diphtheria-pertussis-tetanus (DTP) vaccine, and hepatitis vaccines, all of which have been administered. The mother experienced gestational hypertension and diabetes during pregnancy. The child was born via elective cesarean section at term (birth weight 2.9 kg) and has developed normally. There is no reported family history of genetic diseases, and the parents are in good health.

### 2.2 Examination

Temperature 36.6°C, pulse 96 beats/min, respiration 21 breaths/min, blood pressure 131/84 mmHg; height 163.5 cm (+1SD to +2SD), weight 49.4 kg, normal development (Tanner stage B2P2); abnormal physical examination: pharyngeal congestion, bilateral tonsils Grade I hypertrophy, coarse breath sounds in both lungs, diminished breath sounds in the right lung with slight wet rales audible; abdominal wall reflex (+), bilateral ankle-knee reflex (++), no other neurological reflex abnormalities (Babinski’s sign negative).

Laboratory and Imaging Findings: A chest X-ray revealed pneumonia localized to the right lower lung field. Ultrasound imaging identified bilateral pleural effusion, with the right side measuring 13 mm and exhibiting septation, alongside partial consolidation in both lungs. Echocardiographic assessment demonstrated a trace pericardial effusion without signs of infective endocarditis. Abdominal ultrasound findings indicated right hydronephrosis without evidence of infection, while thyroid ultrasound revealed multiple cystic nodules devoid of infection-related changes. The complete blood count indicated a severe infection, with elevated white blood cell count (19.61×10^9/L), neutrophils (78.4%), and platelets (529×10^9/L), along with an increased C-reactive protein level (142.3 mg/L). Blood biochemical analysis showed gamma-glutamyl transferase (GGT) at 27 U/L, lactate at 5.01 mmol/L, procalcitonin (PCT) at 0.11 ng/ml, and interleukin-6 (IL-6) at 138.3 pg/ml. Venous glucose was elevated at 19.26 mmol/L. Coagulation function tests revealed fibrinogen at 6 g/L and D-dimer at 2.45 mg/L, with subsequent follow-up showing fluctuations peaking at 6.93 mg/L before normalization. Venous blood gas analysis was approximately normal. Glycosylated hemoglobin was significantly elevated at 14.3%, with ferritin at 774.00 ng/mL, insulin at 15.23 pmol/L, and C-peptide at 0.282 nmol/L. Tests for antinuclear antibodies (ANAs) and antineutrophil cytoplasmic antibodies (ANCA) were negative.

Infection-Related Investigations: (1) Hematological System: Blood cultures exhibited no bacterial growth over a period of five days, effectively excluding the presence of hematogenous infection. (2) Urinary Tract: Urine cultures were negative on three separate occasions, thereby ruling out bacterial urinary tract infection. Urinalysis indicated a glucose level of 4+ with negative urine ketones, suggesting inadequate glycemic control. (3) Pulmonary System: Metagenomic analysis of alveolar lavage fluid identified the presence of Streptococcus pneumoniae (sequence number 406) and Rhizopus oligorhizus (sequence number 36), signifying a mixed bacterial-fungal infection. Additionally, sputum next-generation sequencing (nGS) detected elevated concentrations of Moraxella catarrhalis. (4) Oral Examination: The oral assessment revealed ulcers on the mucous membranes of the left upper lip and the right oral corner, covered with a yellowish-white pseudomembrane. (5) Additional Findings: Early renal injury markers included N-acetyl-beta-D-glucosaminidase (NAG) at 19.5 U/L, urinary beta-2 microglobulin (Uβ2-MG) at 0.46 mg/L, alpha-1 microglobulin (α1-MG) at 6.5 mg/L, and urinary creatinine (U-Cr) at 1771.00 μmol/L, with other parameters within normal limits. Stool examination was normal. Electrocardiogram results indicated sinus tachycardia with a heart rate of 96 beats per minute and nonspecific T wave abnormalities. 

### 2.3 Diagnosis and differential diagnosis

**Figure 1:**
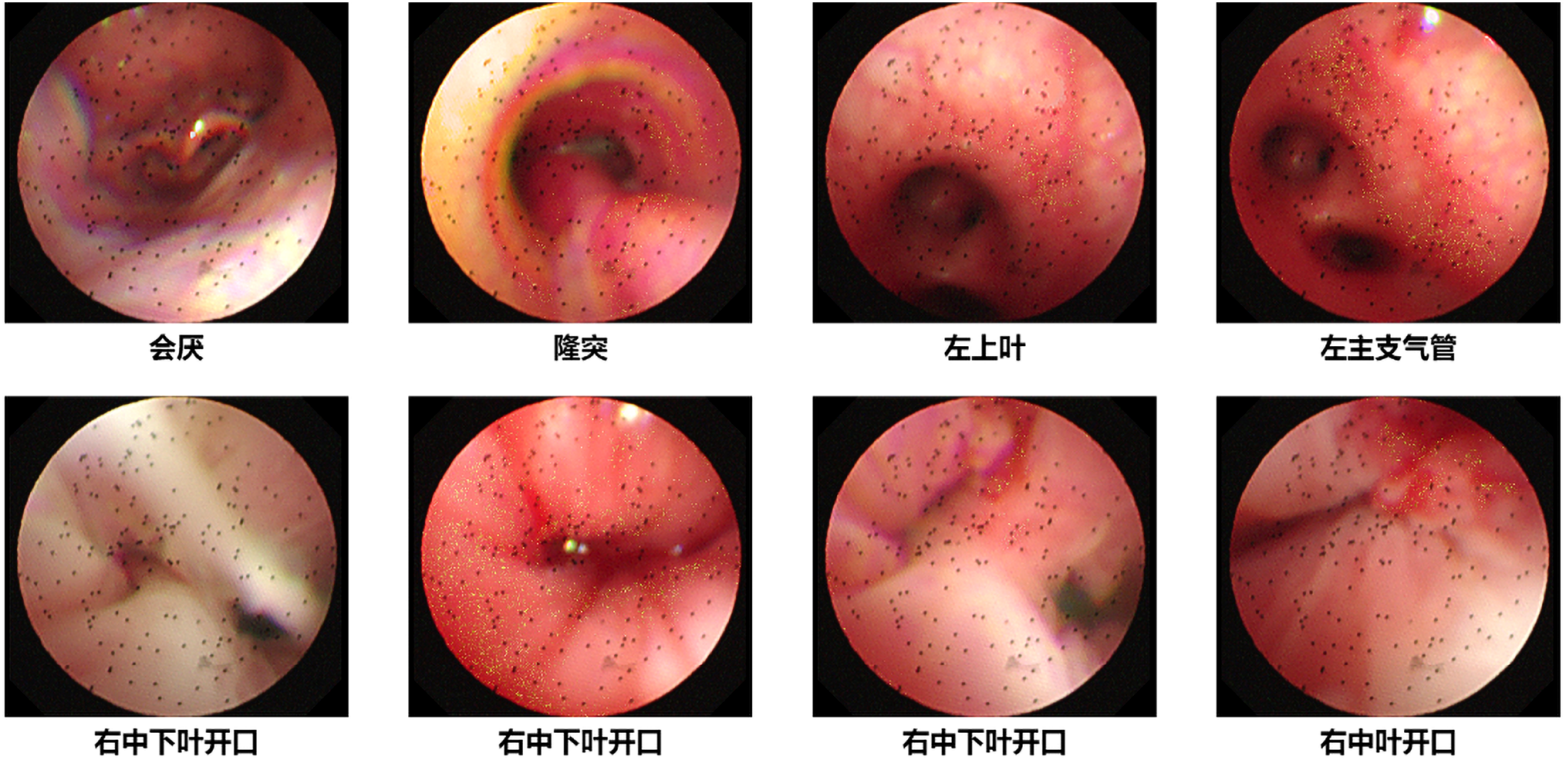
Bronchoscopy examination on January 14, 2025.

Based on comprehensive clinical manifestations, imaging examinations, bronchoscopic findings (**Figure 1**), alveolar lavage fluid, nasopharyngeal swabs, sputum laboratory and pathological examination results, the clinical diagnosis has been established as follows: Severe pneumonia; Pulmonary mucormycosis; Bilateral pleural effusion; Pulmonary abscess to be investigated; Diabetes mellitus; Puberty; Coagulation dysfunction; Lactic acidosis; Pericardial effusion (minimal); Right hydronephrosis; Multiple cystic nodules of the thyroid; Oral thrush; Streptococcus pneumoniae infection.

### 2.4 Treatment

The patient has been diagnosed with diabetes for more than two years and has had a cough for over ten days, being hospitalized eight days ago due to a fever lasting six days. Treatment focused on blood glucose control, anti-infection measures, and management of complications. Upon admission, comprehensive diabetes management was initiated: subcutaneous injection of Novolog (preprandial) and Detemir insulin (at bedtime) for blood glucose control, along with dietary guidance, exercise education, and blood glucose monitoring; on the fourth day, it was adjusted to insulin pump therapy (continuous subcutaneous injection of insulin glargine R) to optimize glucose regulation.

**Figure 2:**
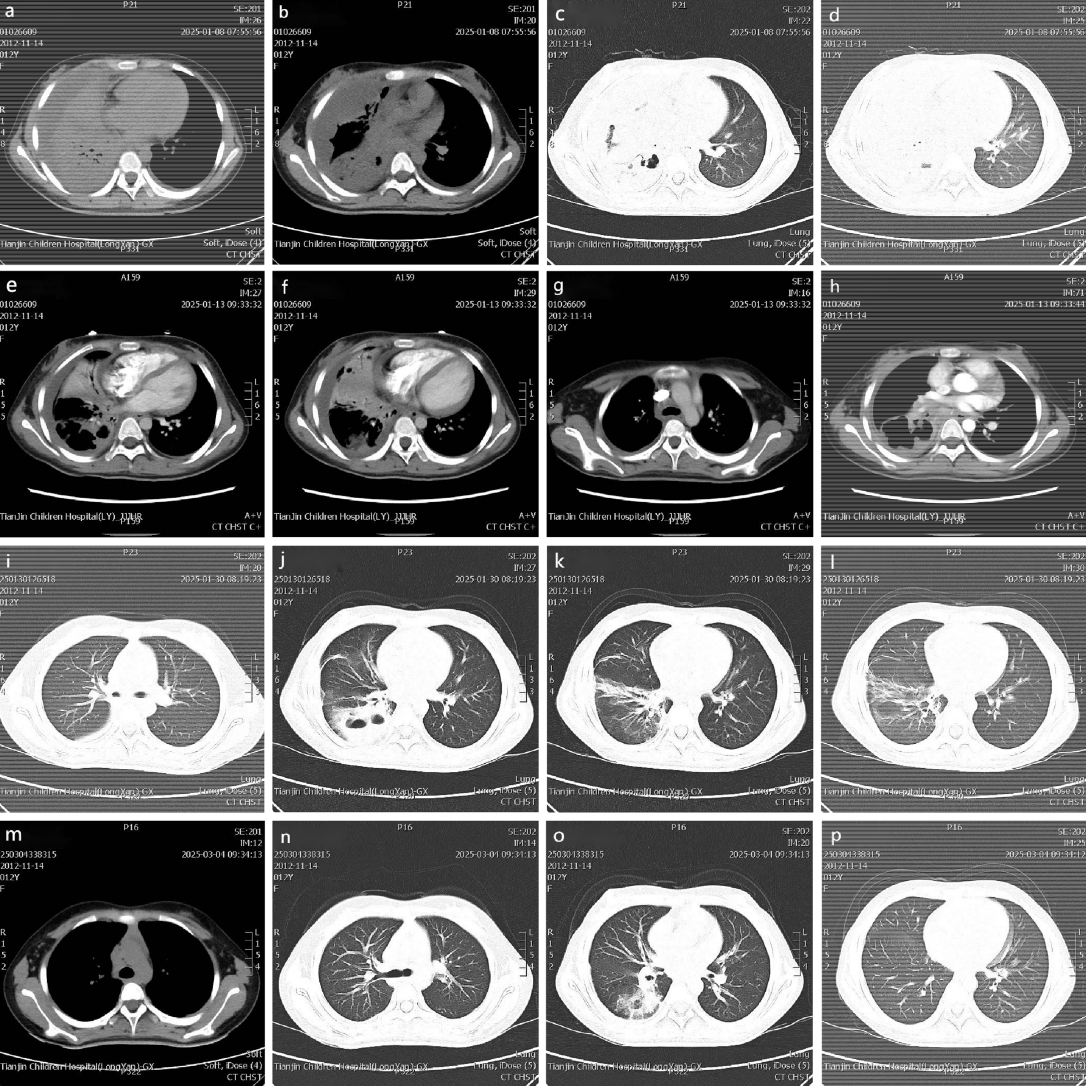
Chest CT results.

During the initial phase of anti-infective treatment, cefotaxime (1g bid) was combined with doxycycline (100mg q12h). However, the patient still experienced recurrent high fever (39°C) along with hypoxemia (blood oxygen saturation 90%-97%), which improved after oxygen supplementation (4L/min). On the second day of hospitalization, lung CT indicated bilateral pulmonary consolidation with atelectasis in the right middle and lower lobes, accompanied by multiple cavities and pleural effusion (**Figures 2A–D**). After adding methylprednisolone (40mg q8h) for anti-inflammatory treatment, the patient’s temperature rapidly returned to normal.

On the fourth day, microbiological testing revealed the presence of Catabacter (normalized sequence number 447189, estimated microbial concentration 1.7×10^5) in the sputum. In conjunction with CT findings and inflammatory markers, concerns for a pulmonary abscess prompted an upgrade in antibiotic treatment to meropenem (1g q8h) combined with linezolid (0.6g q12h), while gradually tapering off methylprednisolone (discontinued on the sixth day) (**Table 1**).

**Table 1:** Sputum nGS results from January 11, 2025.

Results of Pathogenic Microorganism Testing					
Microbial classification	Genus name	Microbial name	Normalized sequence reads	Estimated microbial concentration (copies/ml)	Pathogenicity classification
Bacterial list					
G-	Moraxella	Moraxella catarrhalis	447189	1.70x10^5	A
G+	Parvimonas	Parvimonas micra	2459	620	C
G+	Streptococcus	Streptococcus constellatus	446	83	C
Fungal list					
Fungus	Candida	Candida albicans	270	140	B

On the sixth day, the patient’s condition relapsed, presenting with fever (38.5°C) and oral thrush (oral fungal infection). An enhanced lung CT scan conducted on January 13, 2025, revealed a progression of the large inflammatory consolidation in the right middle lobe compared to the previous scan dated January 10, 2025. This was accompanied by an increase in right pleural effusion with localized encapsulation (**Figures 2E–H**). Bronchoscopy on the eighth day revealed narrowing of the right bronchus with secretions and granulation tissue. Alveolar lavage fluid mNGS detected Streptococcus pneumoniae and a low sequence number of Mucorales (Rhizopus arrhizus) (**Table 2**).

**Table 2:** Alveolar lavage fluid mNGS results from January 17, 2025.

Bacterial List						
Genus				Complex/Species		
Type	Name	Sequence Reads	Relative Abundance	Name	Sequence Reads	Coverage
G+	Streptococcus	1104	98.308%	Streptococcus pneumoniae	406	1.6948%
Fungal List						
Genus				Complex/Species		
Type	Name	Sequence Reads	Relative Abundance	Name	Sequence Reads	Coverage
fun	Rhizopus	61	30.964%	Rhizopus arrhizus	36	0.0065%

**Figure 3:**
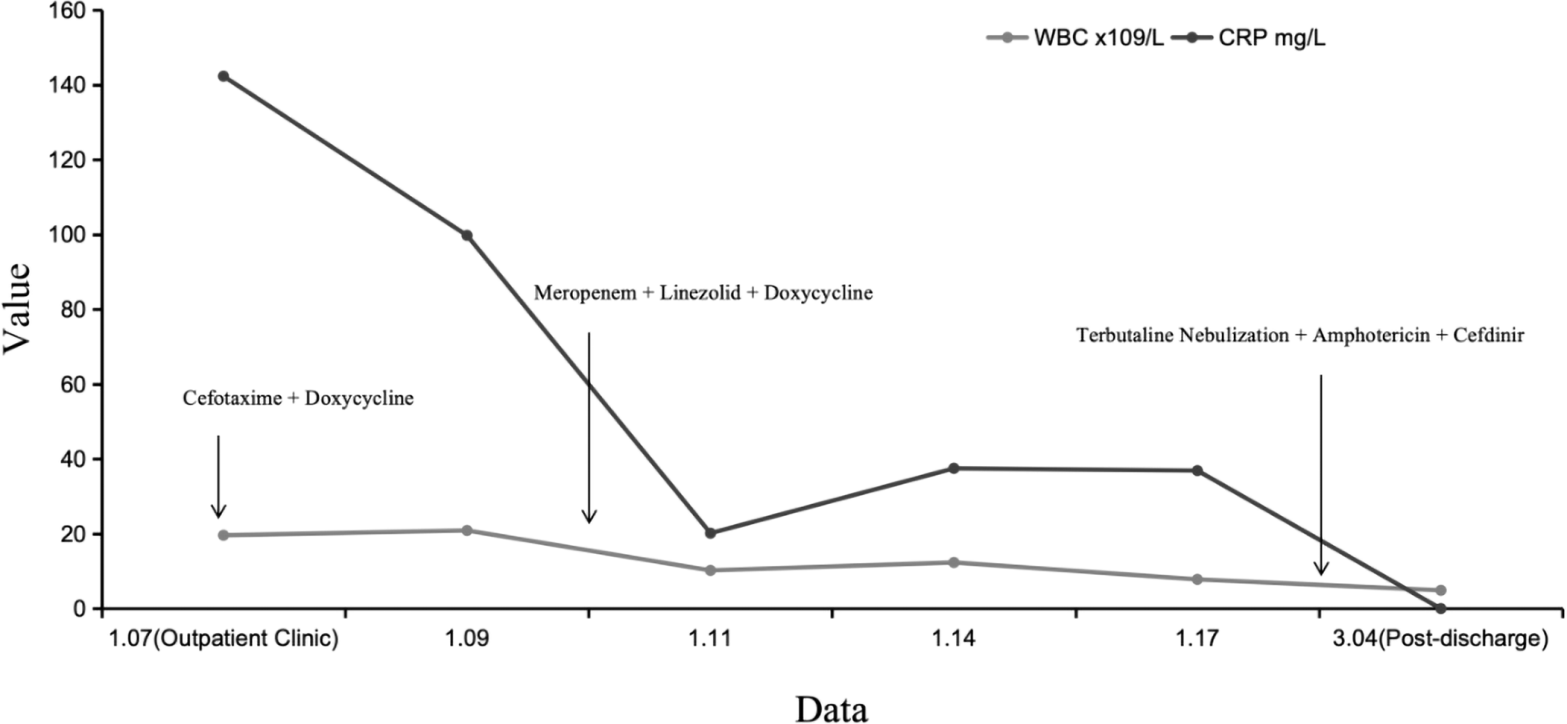
Treatment status. Medications: Cefotaxime (January 7, 2025, to January 10, 2025), Doxycycline (January 7, 2025, to January 14, 2025), Meropenem (January 10, 2025, to January 18, 2025), Linezolid (January 10, 2025, to February 9, 2025), Bricanyl nebulization (January 18, 2025, to February 9, 2025), Amphotericin B (January 18, 2025, to March 4, 2025), Cefdinir (January 18, 2025, to March 4, 2025).

After performing bronchoscopic clearance of secretions, adjusting the anti-infection regimen, and providing supportive treatment, the patient’s temperature and blood oxygen levels returned to normal by the ninth day. Due to the high risk associated with pleural puncture, no intervention was performed, and the patient was discharged stable after 11 days of hospitalization. All medication details during hospitalization are shown in **Figure 3**.

Condition at discharge: Temperature normal, cough present, slightly decreased breath sounds in the right lung, with some moist rales audible. During hospitalization, changes in various hematological parameters are shown in **Table 3**. The initial fasting blood glucose fluctuated between 6.4-17.6 mmol/L, and the postprandial blood glucose (two hours after meals) fluctuated between 6.2-19.1 mmol/L. In the three days prior to discharge, fasting blood glucose fluctuated between 6.1-6.8 mmol/L, while postprandial blood glucose fluctuated between 5.4-11.7 mmol/L.

**Table 3:** Changes of hematological parameters during hospitalization.

Date	Hb g/L	WBC x109/L	N%	L%	M%	PLT x109/L	CRP mg/L
1.07	120	19.61	78.4	15.9	5.6	529	142.3
1.09	118	20.9	87	9	4	485	99.76
1.11	125	10.22	75	21	4	750	20.17
1.14	115	12.34	76	19	5	592	37.49
1.17	111	7.82	67	26	7	370	36.9
3.04	139	4.9	55.2	36.1	5.9	245	<0.5

Chest CT scan findings at admission from January 8, 2025 (a-d); Chest contrast-enhanced CT findings and progression during treatment from January 13, 2025 (e-h); CT review findings and absorption status at mid-treatment from January 30, 2025 (i-l); CT findings and final outcome at the last follow-up from March 4, 2025 (m-p).

### 2.5 Treatment outcomes, follow-up, and prognosis

After discharge, the anti-infection and blood glucose management plan was determined through communication between the medical staff and the patient’s family: 1) For pulmonary mucormycosis, the family agreed to nebulized amphotericin B treatment, initially combining it with 1 vial of Terbutaline (Bricanyl) + 10ml of 0.9% NaCl for nebulization twice daily until February 9, 2025. It was later adjusted to nebulized amphotericin B (2.5mg twice daily on the first day, then 5mg twice daily), but the family independently reduced the nebulization frequency to once daily, not strictly adhering to the bid regimen; 2) The treatment for bacterial infection continued with oral Cefdinir 100mg three times daily (total course of 7 weeks) and oral linezolid 0.6g every 12 hours (continued for 3 weeks post-discharge, total course of 1 month); 3) Blood glucose control was adjusted to subcutaneous injection of NovoRapid (81U before breakfast, 8IU before lunch, 8IU before dinner) and Detemir insulin (20IU at bedtime), with dosage adjusted based on blood glucose monitoring, supplemented with methylcobalamin 1 tablet daily for neuroprotection. 

Three follow-up visits post-discharge (January 30, 2025; February 11, 2025; March 4, 2025) showed: cough gradually diminished, occurring only occasionally after 1.5 months, with normal complete blood counts indicating good infection control; The progression of chest imaging findings is detailed as follows: A subsequent CT scan on January 30, 2025, indicated partial resolution of the scattered inflammatory consolidation in the right lung and a reduction in the right pleural effusion (**Figures 2I–L**). The final follow-up chest CT scan, performed on March 4, 2025 (**Figures 2M–P**), demonstrated further absorption of the scattered inflammatory consolidation in the right lung, an increase in the volume of the right middle and lower lobes relative to pre-admission measurements, a reduction in the extent of multiple cavitation lesions in the right lower lobe, continued absorption of the right pleural effusion, and improvement in bilateral pleural thickening compared to pre-admission. However, partial obstruction of the right bronchus persisted; fasting blood glucose stabilized between 6-7mmol/L, and 2-hour postprandial glucose was between 7-9mmol/L, with total insulin dosage reduced compared to the hospitalization period (from 20IU to 10IU for Detemir), indicating metabolic improvement. The patient continues to have regular follow-ups, with the next appointment scheduled for one month later. **Table 4** provides a summary of the key details and timeline of this treatment process.

**Table 4:** Summary of key details and timeline.

Time Point	Event or Key Details
Two years ago	The patient was diagnosed with type 1 diabetes and initiated long-term insulin therapy, including aspart and detemir insulin.
10 days prior to admission	The patient began experiencing a cough accompanied by mucus.
8 days prior to admission	The patient developed a fever, with a maximum temperature reaching 40 °C.
Before admission	The patient received treatment with dexamethasone, clindamycin, and ambroxol at an external facility; however, symptoms of fever subsided without complete control of the condition.
January 7, 2025	The patient was admitted due to a cough lasting more than 10 days and a fever persisting for 6 days.
At admission	Physical examination revealed pharyngeal congestion, bilateral grade I enlargement of the tonsils, coarse breath sounds in both lungs, diminished breath sounds in the right lung, and some moist rales; laboratory tests indicated signs of a severe infection.
1 day after admission	Comprehensive diabetes management was commenced, involving subcutaneous injections of Novolog (before meals) and detemir insulin (before bedtime), in conjunction with dietary guidance, exercise education, and blood glucose monitoring.
2 days after admission	Chest CT showed consolidation in both lungs with atelectasis in the right middle and lower lobes, as well as multiple cavities and pleural effusion.
4 days after admission	The insulin pump therapy was adjusted (continuous subcutaneous injection of insulin glargine R) to optimize blood glucose regulation; sputum microbiological testing revealed Catabacter.
6 days after admission	The patient’s condition relapsed, presenting with a fever (38.5 °C) and oral candidiasis (oral fungal infection).
8 days after admission	Bronchoscopy revealed narrowing of the right bronchus, with secretions and granulation tissue; mNGS of bronchoalveolar lavage fluid detected Streptococcus pneumoniae and a few other fungi (Mucorales, with a low sequence count).
9 days after admission	Following bronchoscopy secretion clearance, adjustments to the anti-infective regimen, and supportive treatment, the patient’s temperature and blood oxygen levels returned to normal.
At discharge	The patient was discharged in a stable condition after a total of 11 days in the hospital.
Post-discharge (January 18 - 30)	An anti-infection and blood glucose management plan was established: nebulization with amphotericin B continued for the treatment of pulmonary mucormycosis, oral Cefdinir and oral Linezolid were used for bacterial infection treatment, and insulin dosage was adjusted.
January 30, 2025	During the first follow-up, the patient’s cough gradually diminished, and blood test indicators showed good control of the infection.
February 11, 2025	During the second follow-up, the X-ray indicated an improvement in the pneumonia and a resolution of the right pleural effusion. A patchy high-density shadow in the middle zone of the right lung, with an irregular low-density shadow visible within it, and the extent has decreased. Both hilar shadows appear blurred
March 4, 2025	During the third follow-up, chest CT indicated partial absorption of scattered inflammatory consolidation in the right lung, with improved volume in the right middle and lower lobes and a reduced extent of multiple cavitary lesions in the right lower lung the patient experienced occasional cough, stable blood glucose levels, and a reduced total insulin dosage compared to that during hospitalization.

## 3 Discussion

This case report describes the diagnosis and treatment process of a 12-year-old patient with diabetes complicated by pulmonary mucormycosis. The clinical complexity of this case is reflected in multiple infections, metabolic disorders, and challenges in treatment adherence. The following discussion addresses key issues in conjunction with relevant literature.

### 3.1 The correlation between pulmonary mucosal fungal infections and the administration of hormonal treatments

The patient has a significant history of using hormone medications. While it is not possible to definitively assert that the fungal infection was exclusively attributable to hormonal influences, we cannot entirely dismiss the potential correlation. A thorough evaluation of the mechanisms by which hormones operate, the patient’s immune status, and the timing and context of the infection is warranted. The potential relationship between hormonal administration and fungal infections is primarily based on the significant immunosuppressive effects of these hormones, which can attenuate inflammatory responses and impair immune function (7). In this instance, the patient was administered intravenous dexamethasone followed by methylprednisolone as part of her anti-inflammatory treatment regimen. It is well-established that individuals with diabetes often exhibit compromised immune responses, rendering them more susceptible to infections (8). The administration of hormones may further diminish the patient’s immune defenses, thereby increasing vulnerability to opportunistic pathogens such as fungi. Notably, the patient exhibited fever and cough shortly after the initiation of hormonal therapy, which ultimately culminated in a diagnosis of mucormycosis. Thus, hormonal treatment likely acted as a significant trigger or exacerbating factor in the development of this infection. The condition was managed effectively following the administration of antibiotic and antifungal therapies, such as amphotericin B, demonstrating the infection’s responsiveness to treatment and indirectly implying its presence and severity. The progressive reduction of hormonal therapy, followed by antifungal treatment, likely played a significant role in the observed clinical improvement.

### 3.2 The association between adolescent diabetes and pulmonary mucormycosis

Pulmonary mucormycosis is predominantly observed in immunocompromised individuals or those experiencing diabetic ketoacidosis (DKA). However, instances of such infections in adolescents with diabetes are infrequently reported. In this particular case, the patient exhibited persistently poor glycemic control, as evidenced by an HbA1c level of 14.3%. The immune dysfunction and acidic microenvironment associated with hyperglycemia may have contributed to the proliferation of mucormycetes (8). Studies have indicated that abnormal iron metabolism in diabetic patients, such as hyperferritinemia, can further elevate the risk of mucormycosis (9). In this patient, the serum ferritin level was significantly elevated at 774 ng/mL, aligning with findings in the literature. Adolescents with diabetes experience complex immune regulatory mechanisms due to physiological development and hormonal fluctuations, necessitating heightened vigilance for opportunistic infections (10).

### 3.3 Challenges of pathogen diagnosis and the value of mNGS technology

In this case, early empirical antibiotic treatment (cefotaxime + doxycycline) failed to control the condition. Ultimately, bronchoalveolar lavage fluid mNGS detected a low number of Rhizopus species (low sequence count) and Streptococcus pneumoniae, confirming a mixed infection. Traditional culture methods have a low detection rate for mold (approximately 30%) and are time-consuming (11). In contrast, mNGS can quickly identify rare or low-abundance pathogens, which is especially valuable for diagnosis in immunocompromised patients (12). However, when the sequence count is low, careful interpretation in conjunction with clinical findings is necessary to avoid overdiagnosis (13).

### 3.4 Antifungal treatment strategies and compliance issues

Amphotericin B is the first-line drug for mucormycosis (17), but intravenous administration is limited by nephrotoxicity and tolerability in children (18). In this case, nebulized amphotericin B (5 mg bid) combined with systemic antibacterial treatment achieved certain efficacy; however, the family’s decision to reduce the frequency of nebulization may impact long-term outcomes. Studies indicate that nebulized amphotericin B can enhance local drug concentration and reduce systemic side effects, but it demands high compliance (14). Furthermore, insufficient treatment duration may lead to relapse, necessitating enhanced patient education and development of personalized follow-up plans (15).

### 3.5 Correlation between blood glucose control and infection prognosis

During the patient’s hospitalization, fasting blood glucose fluctuated between 6.1-17.6 mmol/L. After discharge, glucose levels stabilized at 6–7 mmol/L through intensive insulin therapy, with concurrent improvement in infection markers. Hyperglycemia can exacerbate infections by impairing neutrophil function and promoting the release of inflammatory factors (16), while strict blood glucose control (target HbA1c <7%) can significantly reduce infection-related mortality (19). This case underscores the importance of multidisciplinary collaboration (between endocrinology and infectious diseases) in managing complex cases.

### 3.6 Limitations

The limitations of this case include: ① Lack of histopathological diagnosis (due to the high risk of thoracentesis); ② The dosage and duration of nebulized amphotericin B lack support from pediatric standard guidelines; ③ Limited long-term follow-up data. Future studies should accumulate more pediatric cases to optimize treatment strategies.

## 4 Conclusion

In this case, a 12-year-old patient with diabetes and pulmonary mucormycosis was rapidly diagnosed with a Rhizopus infection through bronchoalveolar lavage fluid mNGS technology. Treatment with nebulized amphotericin B combined with intensive insulin therapy successfully controlled the infection and stabilized blood glucose levels. The case highlights: (1) Adolescents with diabetes should be vigilant for rare fungal infections due to prolonged hyperglycemia and immune dysregulation; (2) mNGS technology has significant diagnostic value for mixed infections and low-abundance pathogens; (3) Multidisciplinary collaboration and personalized treatment are key to improving prognosis. Future attention should be given to the standardization of nebulized antifungal protocols in children and long-term follow-up data to optimize treatment strategies.

## Data Availability

The original contributions presented in the study are included in the article/supplementary material. Further inquiries can be directed to the corresponding author.

## References

[1] Jeong W Keighley C Wolfe R Lee WL Slavin MA Kong DCM . The epidemiology and clinical manifestations of mucormycosis: a systematic review and meta-analysis of case reports. Clin Microbiol infection. (2019) 25:26–34. doi: 10.1016/j.cmi.2018.07.011, PMID: 30036666

[2] Jeong W Keighley C Wolfe R Lee WL Slavin MA Kong DCM . Mucormycosis in COVID-19: a systematic review of cases reported worldwide and in India. Diabetes Metab Syndrome: Clin Res Rev. (2021) 15:102146. doi: 10.1016/j.dsx.2021.05.019, PMID: 34192610 PMC8137376

[3] Otto WR Pahud BA Yin DE . Pediatric mucormycosis: a 10-year systematic review of reported cases and review of the literature. J Pediatr Infect Dis Soc. (2019) 8:342–50. doi: 10.1093/jpids/piz007, PMID: 31181136

[4] Chen Y Feng W Ye K Guo L Xia H Guan Y . Application of metagenomic next-generation sequencing in the diagnosis of pulmonary infectious pathogens from bronchoalveolar lavage samples. Front Cell infection Microbiol. (2021) 11:541092. doi: 10.3389/fcimb.2021.541092, PMID: 33777827 PMC7991794

[5] Zhang Y Wei E Niu J Yan K Zhang M Yuan W . Clinical features of pediatric mucormycosis: role of metagenomic next generation sequencing in diagnosis. Front Cell Infection Microbiol. (2024) 14:1368165. doi: 10.3389/fcimb.2024.1368165, PMID: 38915923 PMC11194326

[6] Walsh TJ Gamaletsou MN McGinnis MR Hayden RT Kontoyiannis DP . Early clinical and laboratory diagnosis of invasive pulmonary, extrapulmonary, and disseminated mucormycosis (zygomycosis). Clin Infect Dis. (2012) 54:S55–60. doi: 10.1093/cid/cir868, PMID: 22247446

[7] Lionakis MS Kontoyiannis DP . Glucocorticoids and invasive fungal infections. Lancet. (2003) 362:1828–38. doi: 10.1016/S0140-6736(03)14904-5, PMID: 14654323

[8] García-Carnero LC Mora-Montes HM . Mucormycosis and COVID-19-associated mucormycosis: insights of a deadly but neglected mycosis. J Fungi. (2022) 8:445. doi: 10.3390/jof8050445, PMID: 35628701 PMC9144279

[9] Ibrahim AS Spellberg B Walsh TJ Kontoyiannis DP . Pathogenesis of mucormycosis. Clin Infect Dis. (2012) 54:S16–22. doi: 10.1093/cid/cir865, PMID: 22247441 PMC3286196

[10] Dannaoui E . Molecular tools for identification of Zygomycetes and the diagnosis of zygomycosis. Clin Microbiol Infection. (2009) 15:66–70. doi: 10.1111/j.1469-0691.2009.02983.x, PMID: 19754761

[11] Wang C You Z Fu J Chen S Bai D Zhao H . Application of metagenomic next-generation sequencing in the diagnosis of pulmonary invasive fungal disease. Front Cell infection Microbiol. (2022) 12:949505. doi: 10.3389/fcimb.2022.949505, PMID: 36237437 PMC9551268

[12] Cornely OA Alastruey-Izquierdo A Arenz D Chen SCA Dannaoui E Hochhegger B . Global guideline for the diagnosis and management of mucormycosis: an initiative of the European Confederation of Medical Mycology in cooperation with the Mycoses Study Group Education and Research Consortium. Lancet Infect Dis. (2019) 19:e405–21. doi: 10.1016/S1473-3099(19)30312-3, PMID: 31699664 PMC8559573

[13] Hoenigl M Salmanton-García J Walsh TJ Nucci M Neoh CF Jenks JD . Global guideline for the diagnosis and management of rare mould infections: an initiative of the European Confederation of Medical Mycology in cooperation with the International Society for Human and Animal Mycology and the American Society for Microbiology. Lancet Infect Dis. (2021) 21:e246–57. doi: 10.1016/S1473-3099(20)30784-2, PMID: 33606997

[14] Godet C Cateau E Rammaert B Grosset M Le Moal G Béraud G . Nebulized liposomal amphotericin B for treatment of pulmonary infection caused by hormographiella aspergillata: case report and literature review. Mycopathologia. (2017) 182:709–13. doi: 10.1007/s11046-017-0117-9, PMID: 28144821

[15] ElSayed NA Aleppo G Aroda VR Bannuru RR Brown FM Bruemmer D . 16. Diabetes care in the hospital: standards of care in diabetes-2023. Diabetes Care. (2023) 46:S267–78. doi: 10.2337/dc23-S016, PMID: 36507644 PMC9810470

[16] Jafar N Edriss H Nugent K . The effect of short-term hyperglycemia on the innate immune system. Am J Med Sci. (2016) 351:201–11. doi: 10.1016/j.amjms.2015.11.011, PMID: 26897277

[17] Al-Azzam N Elsalem L Abu Mousa BM . Population Study Insights Linked to Genetic Variants (rs16890979 and rs206833) and Type 2 Diabetes Control in Northern Jordan. BIOI. (2024) 5. doi: 10.15212/bioi-2024-0005

[18] Ding Y Wei J Ji E Wang M Wu Y Chang M . Improvement of myocardial fibrosis injury by Shengmai injection in ischemia-induced heart failure in a rat model. Sci Tradit Chin Med. (2024) 2:245–253. doi: 10.1097/st9.0000000000000040

[19] Hao J Ye Y Zhang G Shen H Li J Chen G . Mechanisms of nitric oxide in spinal cord injury. Med Gas Res. (2024) 14:192–200. doi: 10.4103/mgr.MEDGASRES-D-23-00006, PMID: 39073327 PMC11257186

